# A Review of Luminescent Anionic Nano System: d^10^ Metallocyanide Excimers and Exciplexes in Alkali Halide Hosts

**DOI:** 10.3390/ma6072595

**Published:** 2013-06-25

**Authors:** Xiaobo Li, Howard H. Patterson

**Affiliations:** 1 Department of Chemistry, University of Maine, Orono, ME 04469, USA; E-Mail: xli@chm.uri.edu; 2 Department of Chemistry, University of Rhode Island, Kingston, RI 02881, USA

**Keywords:** excimers, exciplexes, d^10^ cyanide systems, alkali halide nano systems, dimmers, trimers

## Abstract

Dicyanoaurate, dicyanoargentate, and dicyanocuprate ions in solution and doped in different alkali halide hosts exhibit interesting photophysical and photochemical behavior, such as multiple emission bands, exciplex tuning, optical memory, and thermochromism. This is attributed to the formation of different sizes of nanoclusters in solution and in doped hosts. A series of spectroscopic methods (luminescence, UV-reflectance, IR, and Raman) as well as theoretical calculations have confirmed the existence of excimers and exciplexes. This leads to the tunability of these nano systems over a wide wavelength interval. The population of these nanoclusters varies with temperature and external laser irradiation, which explains the thermochromism and optical memory. DFT calculations indicate an MLCT transition for each nanocluster and the emission energy decreases with increasing cluster size. This is in agreement with the relatively long life-time for the emission peaks and the multiple emission peaks dependence upon cluster concentration.

## 1. Introduction

The term “excimer” was first introduced in 1960 by Stevens and Hutton when studying pyrene luminescence [1]. They defined excimer as a dimer associated in the electronic excited state and dissociated in its ground state, which is different from a stable dimer. Actually, the first excimer can be dated back to 1927, when Lord Rayleigh observed the ultraviolet emission spectrum of high-pressure mercury vapor [2]. Nobel gases, such Helium, Neon, Argon, and Krypton also form excimers to give intense emission spectra in the vacuum ultraviolet region [3,4]. Excimers are also common in liquid and solid phases, especially for aromatic compounds. As early as the 1950s, Förster and Kasper observed that the pyrene solution fluorescence depended on the concentration [5,6]. Since then, more and more organic compounds have been found to form excimers: benzene, naphthalene, 1,2-benzanthracene, and perylene, just to name a few [7,8,9,10,11].

For inorganic excimers, a matrix isolation study has been carried out for alkali metals, alkaline earth metals and group VIIB metals with absorption and luminescence spectra [12]. Platinum complexes have been reported to form different excimers in aqueous solution, such as [PtII(4,7-diphenyl-1,10-phenanthroline)(CN)_2_], [Pt_2_(P_2_O_5_H_2_)_4_^2−^Tl], and [Pt_2_(P_2_O_5_H_2_)_4_^2−^Au(CN)_2_^−^] [13,14,15]. In aqueous solution, the formation of excimers is affected by the concentration of monomers; as the concentration increases, the emission peak intensity corresponding to the excimer is enhanced. Closed-shell heavy metals used to be considered as repelling each other due to the fully occupied valence orbitals. However, in the study of inorganic and organometallic compounds, experimental results have presented evidence of interaction between closed-shell metals [16,17,18,19,20,21,22,23,24,25]. The strength of these bonds is weaker than ionic (700–4000 kJ/mol) and covalent bonds (200–1000 kJ/mol) but stronger than a van der Waals bond (<5 kJ/mol), and they are about the energy of hydrogen bonds (10–40 kJ/mol) [26]. The strong aurophilic interaction is ascribed to the relativistic effect of gold. In chemistry, relativistic effect means when the electrons with high speed move close to a heavy nucleus, the mass increases consequently, and so does the effective nuclear charge [27]. Thus the less diffuse orbitals (s and p) contract radially and the orbital energy is stabilized, leading to a stronger shielding of the nuclear attraction. The more diffuse orbitals (d and f) expand radially and results in a destabilization of the orbital energy. Gold has a larger relativistic effect than any other element with *Z* < 100 [28]. The tendency of Au(I) to form larger clusters via a weak interaction is due to the stabilization of molecular orbitals derived from the filled 5d atomic orbitals by configuration mixing with empty molecular orbitals from 6s or 6p atomic orbitals [29].

Inspired by the Au(I) compounds, our group has reported the first example of ligand-unsupported Ag(I) complex with argentophilic interaction, Tl[Ag(CN)_2_] [30]. X-ray crystallography, luminescence, and temperature-dependent Raman spectra measurements were carried out. The crystal structure results show that the Ag–Ag bond distance is 3.11 Å and the low energy Ag–Ag vibrational frequency of about 75–125 cm^−1^ given by Raman indicates the presence of direct argentophilic interaction in Tl[Ag(CN)_2_]. Since then, our group has reported a series of d^10^ metal cyano compounds in nano systems [31,32,33,34,35,36,37,38,39,40,41]. We found that due to the formation of different size exciplexes, these nano systems show interesting photophysical and photochemical behaviors, such as emission tunability, thermochromism, and optical memory. Luminescence of these systems not surprisingly depends upon the content of cyanometallic compounds, similar to excimers in aqueous solution. In this paper, we review the research work concerning Ag(CN)_2_^−^, Au(CN)_2_^−^, and Cu(CN)_2_^−^ nano systems, which possess interesting luminescence properties.

## 2. Ag(CN)_2_^−^ Excimers and Exciplexes

The photoluminescence of solid state Tl[Ag(CN)_2_] indicates the existence of argentophilicity in Ag(I) compounds [30]. The Ag–Ag interaction is believed to result in the formation of *[Ag(CN)_2_^−^] excimers in the bulky state. We carried out a study focusing on the *[Ag(CN)_2_^−^]*_n_* in the nano state by doping Ag(CN)_2_^−^ in KCl hosts [31,32,33,34,39]. The KAg(CN)_2_/KCl nano systems were prepared by a simple slow evaporation method and the concentration was determined using atomic absorption method.

**Figure 1:**
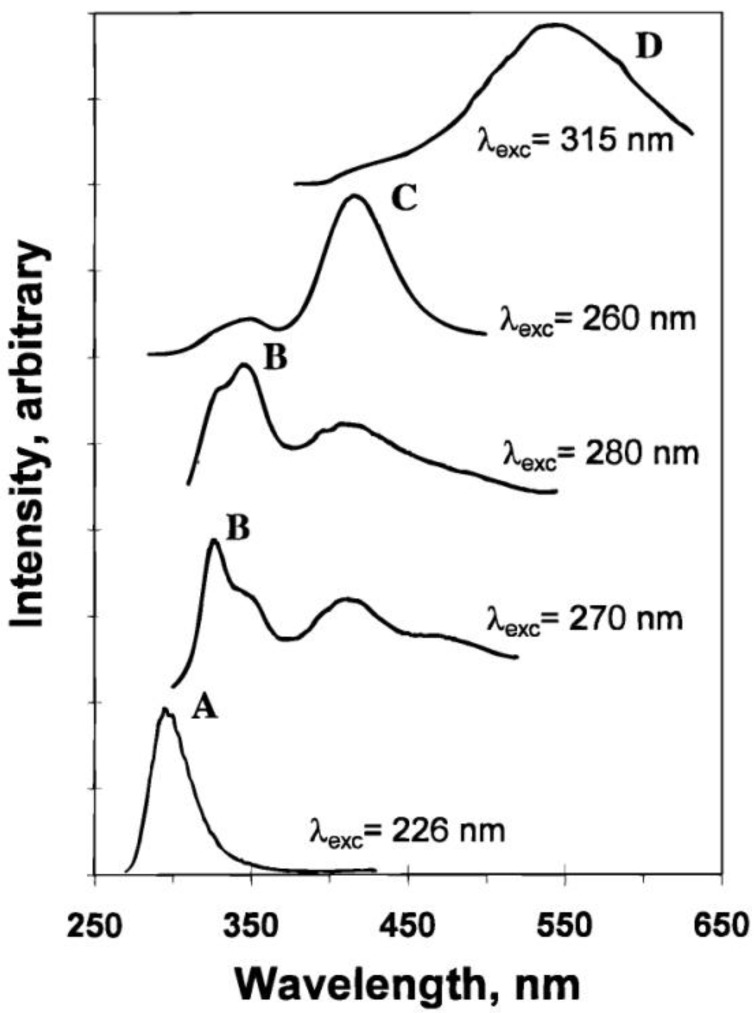
Exciplex tuning by site-selective excitation: emission spectra of a KAg(CN)_2_/KCl crystal at 77 K with different excitation wavelengths. Intensities are not comparable between different spectra. (Reprinted with permission from [31]. Copyright 1998 American Chemical Society.)

Figure 1 shows the emission peaks for the KAg(CN)_2_/KCl single crystal. Multiple emission peaks at 295, 327, 345, 417, and 548 nm were observed for corresponding excitation wavelengths. These luminescence peaks are labeled as A, B, C, and D respectively, as indicated in Figure 1. For each emission band, the corrected excitation spectra were compared to the absorption of a KAg(CN)_2_ aqueous solution. The KAg(CN)_2_ solution absorbs at 196 nm, ascribed to a metal-to-ligand charge transfer process. However, the excitation maxima for A–D bands are at much lower energy and the homometallic interaction is believed to play an important role in the large red-shift of the excitation peaks.

**Figure 2:**
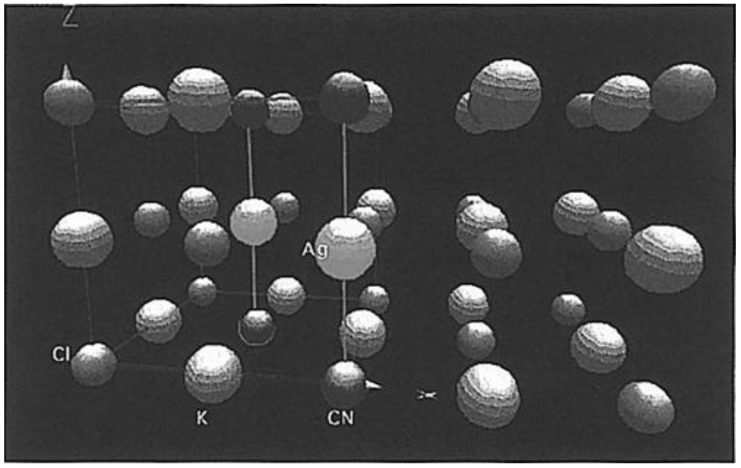
Structure of a unit cell of KCl with a defect created by doping two adjacent Ag(CN)_2_^−^ ions. In the defect, the Ag^+^ and CN^−^ ions are shown to occupy the K^+^ and Cl^−^ sites, respectively. (Reprinted with permission from [31]. Copyright 1998 American Chemical Society.)

To better understand the argentophilic interaction, electronic structure calculations using both extend Hückel and *ab initio* methods were conducted for a monomer, dimer and trimer doped in KCl hosts. As shown in Figure 2, a Ag(CN)_2_^−^ monomer is present in the KCl cubic unit cell with substituting Ag^+^ and CN^−^ for K^+^ and Cl^−^, thus the total charge of this system is unchanged. For dimers, there are three different configurations, eclipsed, perpendicular, and offset-eclipsed, which all have linear Ag(CN)_2_^−^ units. Calculations for four trimers with linear and angular arrangements respectively were carried out as well.

Table 1 summarizes the calculated results for the equilibrium distance, binding energy, HOMO-LUMO gap and overlap population for [Ag(CN)_2_^−^]*_n_* nanoclusters in ground and excited states. The bond distance between two Ag atoms is shorter in the excited state than in the ground state. In addition, the binding energy and the overlap population also increase significantly in the excited states in comparison with the ground states. These are consistent with the behavior of excimer and exciplex. The interaction between monomer units is stronger in the excited state, which results in a stable excited dimer, e.g., excimer. The multiple HOMO-LUMO gap energies by calculation are in agreement with the different luminescence bands (A to D) observed experimentally. Interestingly, the HOMO-LUMO gap show a trend that with an increase in the number of interacting Ag(CN)_2_^−^, the emission energy decreases. This explains the exciplex tuning behavior and the different bands A, B, C, and D are tentatively assigned to excimer, *cis*-localized exciplexs, trans-localized exciplexes, and delocalized exciplexes respectively. The *ab initio* calculation results for all [Ag(CN)_2_^−^]*_n_* nanoclusters indicate a antibonding HOMO to bonding LUMO transition, which is also favorable for the formation of excimers and exciplexes.

**Table 1:** Summary of the Results of Extended Hückel Calculations for the Ground and Excited States of Oligomeric Species of Dicyanoargentate(I). (Adapted with permission from [31]. Copyright 1998 American Chemical Society.)

Species	[S]	[S]_2_	*[S]_2_	[S]_2_	*[S]_2_	[S]_3_	*[S]_3_	[S]_3_	*[S]_3_	[S]_3_	*[S]_3_
		(*ec*)	(*ec*)	(*st*)	(*T1*,*st*)	(*T1*,*ec*)	(*T1*,*ec*)	(*T1*,*st*)	(*T1*,*st*)	(*T1*,*ec*)	(*T1*,*ec*)
Ag–Ag eq. diet, A	8.00*^d^*	3.58	3.00	2.88	2.39	3.49	3.09	2.79	2.46	3.54	3.15
Binding energy, eV	0.00	0.13	1.12	0.22	1.32	0.33	1.47	0.61	2.00	0.29	1.14
H–L gap, eV	4.95	4.37	3.98	4.28	4.13	4.00	3.55	3.73	3.46	4.17	3.83
O.P. (Ag–Ag)	0.000	0.003	0.034	0.069	0.089	−0.008	0.027	0.039	0.079	0.003	0.048

The Ag(CN)_2_^−^/KCl nano system has an extreme wide luminescence tuning range over 18,000 cm^−1^, compared to some other systems [42,43,44]. This new optical behavior is referred to as exciplex tuning, in which the exciplex means excited state oligomers, not limited to dimers. The photoluminescence of the crystal can also be tuned by the content of the Ag in the host [32]. The higher energy bands are predominant for lower silver concentration whereas the spectra for higher silver concentration are mostly of lower energy bands. This further confirms the existence of different size nanoclusters in the doped system and the time-resolved luminescence spectra suggest energy transfer processes from dimers to trimers. The increase of trimer luminescence intensity is related to higher statistical population of trimers in Ag concentrated crystals and more acceptors for the energy transfer.

**Figure 3:**
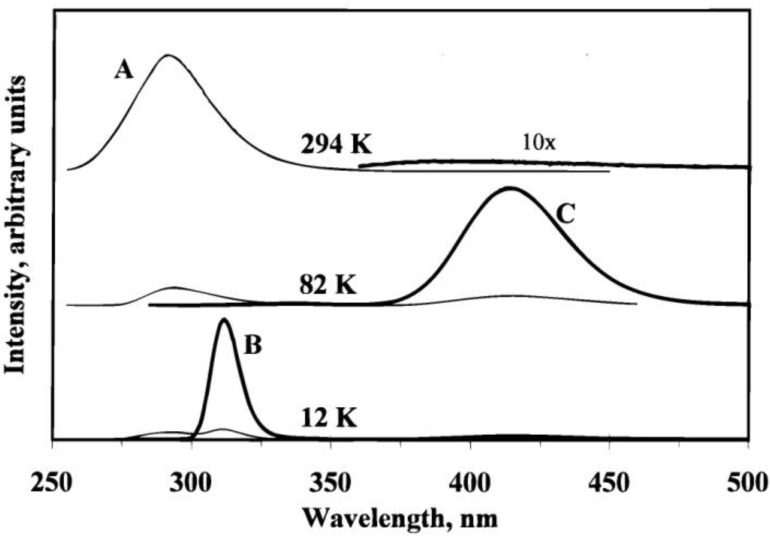
Emission spectra of a KCl/KAg(CN)_2_ crystal as a function of temperature and excitation wavelength. Thin and thick curves represent spectra obtained with excitation wavelengths of 235 and 270 nm, respectively. Intensities are comparable between spectra at the same temperature but not comparable between spectra at different temperatures. (Reprinted with permission from [33]. Copyright 2000 American Chemical Society).

In addition to the novel exciplex tuning behavior, the Ag(CN)_2_^−^/KCl nano system exhibits interesting thermochromism phenomena [33]. Figure 3 shows the emission spectra of a KCl/KAg(CN)_2_ crystal at different temperatures. At room temperature, only one band (A) is observed while excited at 235 nm. However, as the temperature is cooled down to liquid nitrogen temperature (~77 K), band A almost disappears and a strong emission band C at lower energy appears. As the temperature is further decreased to 12 K, the emission spectra show predominantly band B at intermediate energy. Since the luminescence pattern changes dramatically with temperature, the possible cause relating to the Ag–Ag bond distance change is excluded. Another possibility that these emission bands are dependent on fluorescence and phosphorescence is also ruled out, because all the emission bands have a lifetime of microsecond magnitude. Therefore, the thermochromism behavior is due to some major structural changes. A kinetic model based on “normal” and back energy transfer is proposed. At liquid helium temperature, no energy transfer is involved and the luminescence is only from direct excitation. With temperature increasing to 80 K, “normal” energy transfer processes from high energy nanoclusters to low energy ones prevail and thus band C intensity increases in this temperature range. However, a further temperature increase leads to back energy transfer with reverse direction between nanoclusters. The back energy transfer is from low energy to high energy, which is not thermodynamically favorable. The higher activation energy than that of normal energy transfer is compensated by higher temperature, e.g., the back energy is significant at higher temperatures. In addition to KCl, different alkali halides, such as NaCl, NaBr, KBr, and NaF were used to study the host effect. Similar experimental results were obtained except for NaF, which is likely due to the small lattice size leaving no space for the Ag(CN)^−^ clusters.

**Figure 4:**
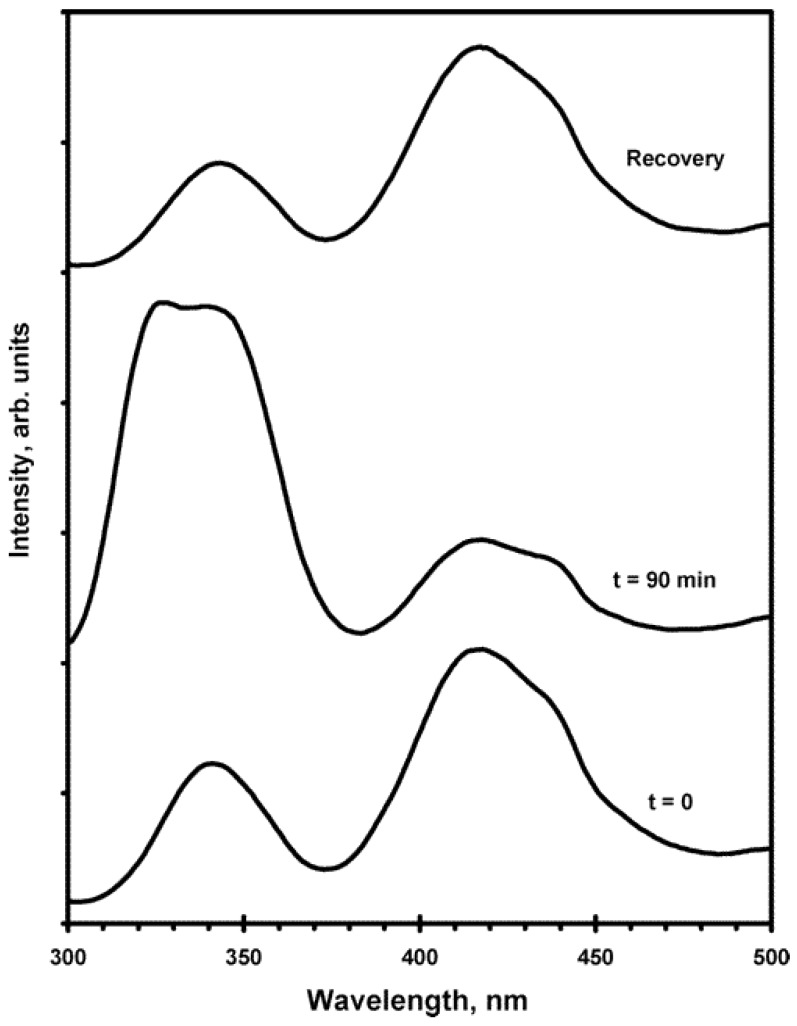
Emission spectra *versus* 266 nm laser irradiation time at 77 K for batch 1 of [Ag(CN)_2_^−^]/KCl crystals. All spectra were scanned with 275 nm excitation. Note the dominance of the short-wavelength bands following irradiation at 77 K and the regeneration of the original spectrum in the recovery step. (Reprinted with permission from [39]. Copyright 2000 American Chemical Society.)

Optical memory is another interesting property resulting from the presence of the nanoclusters in the doped KCl host [39]. As shown in Figure 4, the Ag(CN)_2_^−^/KCl crystal show two emission peaks at 77 K. After irradiation at 266 nm for 90 min, the emission spectra have a significant change with the intensity of high energy peak increasing whereas the low energy peak intensity decreasing. Then, when the crystal is brought up to a warm temperature and cooled down again to 77 K again, the emission pattern recovers back to where it was at the beginning. In the whole process, acquiring emission spectra is “read”, irradiating laser beam on the crystal is “write”, and changing the temperature leads to “erase”. Therefore, this is a reversible write/read/erase optical memory behavior, which is similar to the research done by Zink and coworkers [45,46,47]. During the laser irradiations, the nanoclusters are excited and the distribution of different size excimers and exciplexes is changed, leading to the luminescence intensity change in the emission spectra. The kinetic study was conducted for every five min of laser irradiation and the activation energy was extrapolated via the emission peak intensity *versus* the logarithm of time. The obtained activation energy around 261 J/mol suggests a fast energy transfer between these *[Ag(CN)_2_^−^]*_n_* excimers and exciplexes.

## 3. Au(CN)_2_^−^ Excimer and Exciplexes

**Figure 5:**
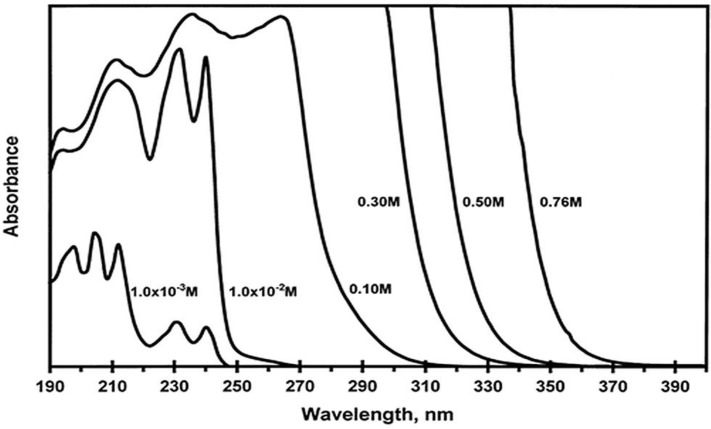
Absorption spectra *versus* concentration of K[Au(CN)_2_] in aqueous solutions at ambient temperature. (Reprinted with permission from [35]. Copyright 2002 American Chemical Society.)

Our group has carried out a study on Ag(CN)_2_^−^ and Au(CN)_2_^−^ in aqueous as well as other organic solutions and observed non-Beer’s law behavior of the absorption for different concentrations of solution, as shown in Figure 5 [35,36]. The luminescence spectra of K[Au(CN)_2_] aqueous solution at room temperature and 77 K exhibit different emission peaks and the pattern depends upon the excitation wavelength. Theoretical calculations indicate the energy gap between the HOMO and LUMO decreases as the oligomer size increases. Both experimental and theoretical results suggest the presence of [Au(CN)_2_^−^]*_n_* excimers and exciplexes. Consequently, investigations on KAu(CN)_2_ doped in an alkali halide host was conducted to further understand the close-shell interaction between Au(I) complexes [38].

**Figure 6:**
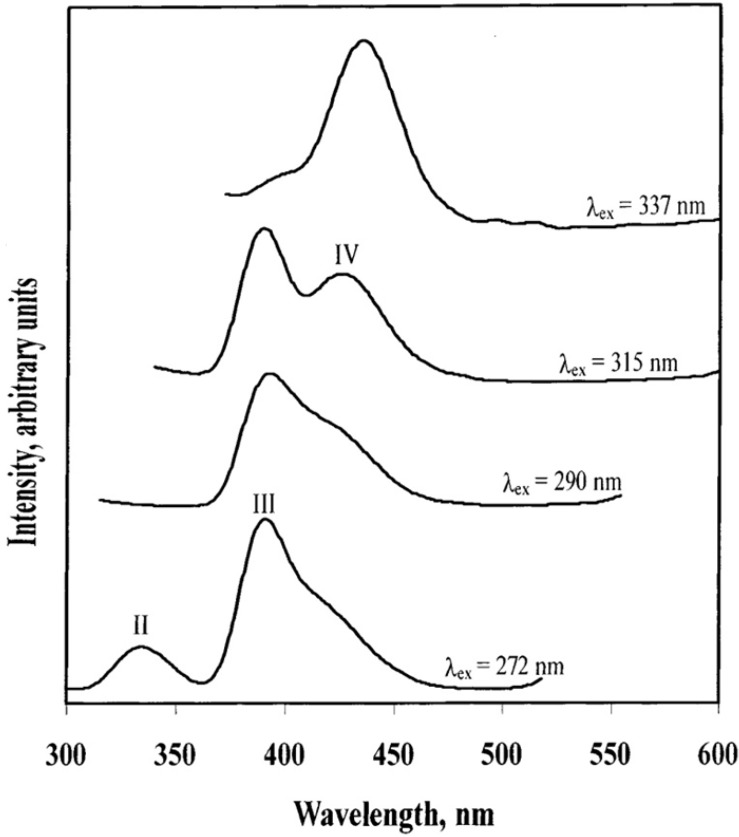
Emission spectra of a single crystal of KAu(CN)_2_/KCl at 77 K with different excitation wavelengths. (Reprinted with permission from [38]. Copyright 2002 American Chemical Society.)

Figure 6 shows the emission spectra of a KAu(CN)_2_/KCl single crystal at 77 K. Same as the argentocyanide/alkali halide system, the Au(CN)_2_^−^/KCl also exhibit exciplex tuning behavior. The excitation ranges from 270 nm to 350 nm and the emission peaks are at 335, 390, and 425 nm respectively. However, the pure KAu(CN)_2_ crystal only shows one emission peak at 390 nm with different excitation wavelengths. Table 2 lists the emission maxima for KAu(CN)_2_ in an alkali halide host and aqueous solution, as well as the corresponding assignments. The high energy emission peak at 275–285 nm in KAu(CN)_2_ aqueous solution was not observed for the solid state, implying no Au(CN)_2_^−^ monomer species is present in the doped KCl crystal. However, for the aqueous solution, due to the limit of concentration, low energy emission peak from the larger size of [Au(CN)_2_^−^]*_n_* does not exist. These results are in agreement with a model where the homometallic interactions between different Au(CN)_2_^−^ units form excimers and exciplexes. The lifetime measurements show microsecond magnitude phosphorescence, suggesting a metal to ligand charge-transfer process. In addition, Raman spectroscopy results provide further evidence for the formation of different nanoclusters in the doped crystal. A pure KAu(CN)_2_ crystal exhibits only one peak in the cyanide stretching region (about 2176 cm^−1^), whereas the doped crystals have additional peaks at 2169 and 2189 cm^−1^. This is consistent with the photoluminescence results. The homometallic interaction in [Au(CN)_2_^−^]*_n_* nanoclusters affects the back bonding from gold to the cyanide ligand, which results in the shift of cyanide stretching frequency.

**Table 2:** General Qualitative Assignment of the Emission Bands Observed in Solids and Solutions of Au(CN)_2_-Species. (Adapted with permission from [38]. Copyright 2002 American Chemical Society.)

band	solids, λ_max_^em^, nm	solutions λ_max_^em^, nm	assignment
I	–	275–285	*[Au(CN)_2_^−^]_2_
II	320–355	320–350	bent *[Au(CN)_2_^−^]_3_
III	370–395	380–390	linear *[Au(CN)_2_^−^]_3_
IV	420–450	420–440	*[Au(CN)_2_^−^]_4_
V	–	455–490	*[Au(CN)_2_^−^]*_n_^a^*
VI	600–640	–	*[Au(CN)_2_^−^]*_n_*

*^a^* *[Au(CN)_2_^−^]*_n_* represents delocalized exciplexes.

Theoretical calculations were conducted using Extended Hückel methods for [Au(CN)_2_^−^] monomer, dimer, trimer, and tetramer. The energy results of the ground state and the first excited-state results for these oligomers show that with the increase of the size, the binding energy between [Au(CN)_2_^−^] units increase and the HOMO-LUMO gap decreases. All the results indicate the bonding between Au-Au is stronger in the excited state and this confirms the existence of [Au(CN)_2_^−^]*_n_* excimers and exciplexes. For the trimer, the geometry also plays an important role and leads to different emission centers. The dynamics of [Au(CN)_2_^−^]*_n_* oligomer geometry changes have been studied by ultrafast spectroscopic experiments [48]. The time-resolved results show direct evidence of a shortening of Au-Au distance and further support the formation of [Au(CN)_2_^−^]*_n_* nanoclusters.

## 4. Cu(CN)_2_^−^ Excimer and Exciplexes

As the last member in the coinage group, copper has a smaller relativistic effect than gold and silver and the homometallic interaction between Cu(I) compounds should be less [28]. Moreover, most of the reported luminescent Cu(I) complexes are cationic with chelating imine ligands or bisphosphine ligands or cuprous clusters [49]. However, our group has reported photophysical and photochemical properties of anionic Cu(CN)_2_^−^ doped in alkali halide hosts which resulted from the close-shell interaction of Cu(I) [41].

**Figure 7:**
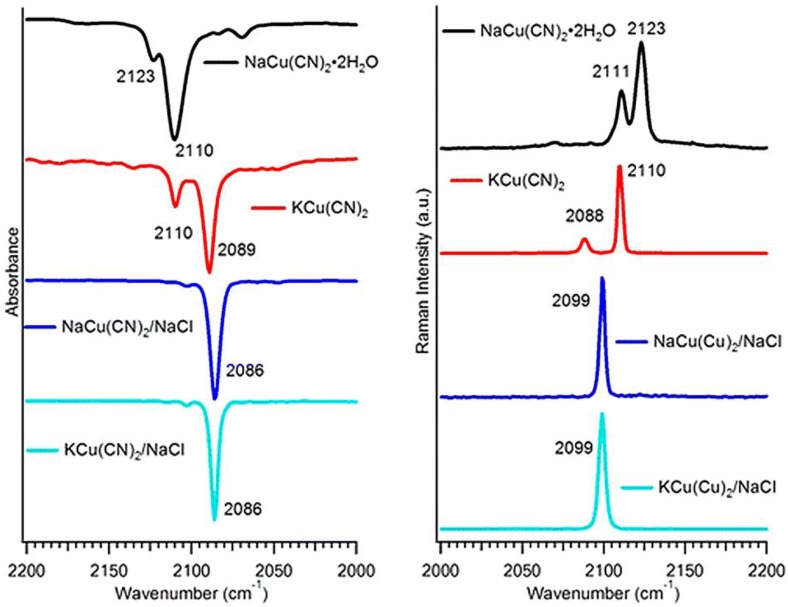
The υ_CN_ region IR and Raman spectra of pure NaCu(CN)_2_·2H_2_O, KCu(CN)_2_ and doped in NaCl. (Reprinted with permission from [41]. Copyright 2012 American Chemical Society.)

Unlike pure KAg(CN)_2_ and KAu(CN)_2_ which have linear M(CN)_2_^−^, the dicyanocuprate salts have a local trigonal Cu(CN)_2_^−^ structure. X-ray diffraction (XRD) results indicate that both NaCu(CN)_2_ 2H_2_O and KCu(CN)_2_ have a polymeric-chain structure. NaCu(CN)_2_ has zigzag CuCN chains linked to [Na(H_2_O)_2_]^+^ polyhedra and the copper has a trigonal-planar structure, with two bridge cyanides for the polymeric chain and one for the [Na(H_2_O)_2_]^+^ polyhedra. However, in KCu(CN)_2_, the polymeric chain is along a two-fold screw axis and the three-coordination of copper is not planar. However, when NaCu(CN)_2_ and KCu(CN)_2_ is doped in alkali halide hosts, the copper(I) cation becomes six-coordinated within the crystal field. With two cyanides and four surrounding halide atoms; the [Cu(CN)_2_X_4_]^5−^ (X for halides) has a local D_4h_ symmetry. Figure 7 gives IR and Raman spectra in the CN stretching region for the pure and NaCl doped dicyanocuprate(I) crystals. The pure NaCu(CN)_2_·2H_2_O, KCu(CN)_2_ have two CN vibrational modes in both IR and Raman, which is due to the local C_2v_ symmetry of trigonal Cu(CN)_2_^−^. For the doped crystals, the same results were observed for both dicyanocuprate salts, suggesting the linear structure in NaCl hosts. Same results were obtained for NaBr and KBr doped Cu(CN)_2_^−^ crystals. This structural change is also confirmed by UV-vis diffuse reflectance and aqueous solution absorption spectroscopy experiments.

**Figure 8:**
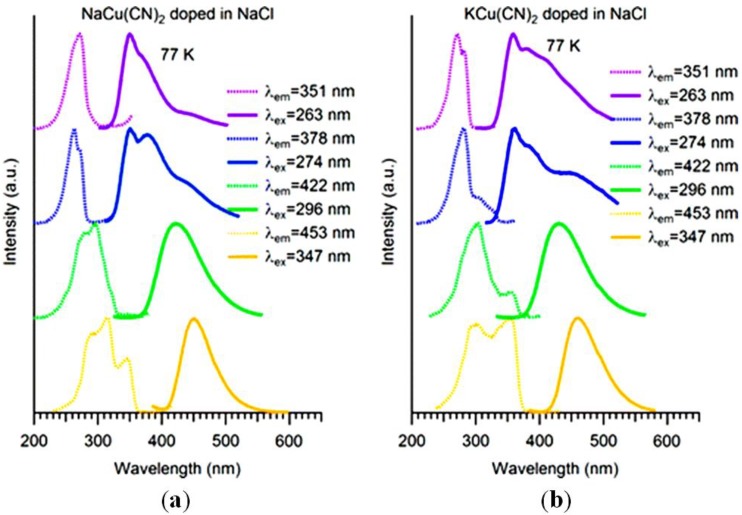
Luminescence spectra at 77 K of (**a**) NaCu(CN)_2_ and (**b**) KCu(CN)_2_ doped in NaCl. (Reprinted with permission from [41]. Copyright 2012 American Chemical Society.)

The Cu(CN)_2_^−^ doped in alkali halide host systems show a series of interesting photophysical and photochemical properties. Similar to Au(CN)_2_^−^ and Ag(CN)_2_^−^, the dicyanocuprate ion in sodium chloride crystal exhibits exciplex tuning, as indicated in Figure 8. Both NaCu(CN)_2_·2H_2_O and KCu(CN)_2_ in NaCl show the same luminescence pattern, which provides further evidence for the linear structure of Cu(CN)_2_^−^ in doped hosts. Other alkali halides, such as NaBr and KBr, were also used and the result was that all the crystals have exciplex tuning. For each host, both NaCu(CN)_2_·2H_2_O and KCu(CN)_2_ give the same luminescence pattern. The intensity of emission peaks depends upon the concentration of the copper in these crystals. The relative peak intensity of low energy peak to high energy peak increases with higher Cu(CN)_2_^−^ content. As we have discussed for Ag(CN)_2_^−^/KCl crystals, the lower energy peaks are assigned to larger size nanoclusters, therefore in the copper case, the increase of Cu(CN)_2_^−^ concentration consequently leads to the formation of more large size oligomers and the lower energy peaks arise.

**Figure 9:**
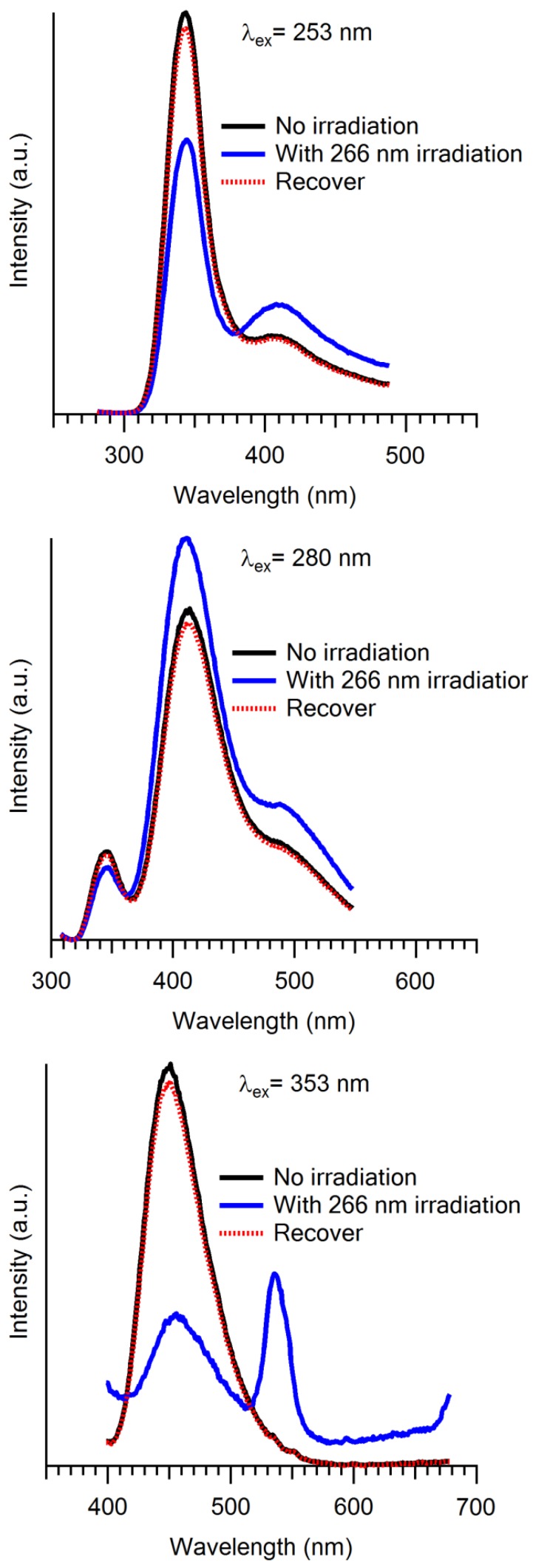
Emission spectra of Cu(CN)_2_^−^ doped in KBr at 77 K without laser irradiation (black solid), with 266 nm laser irradiation (blue solid), and recover, e.g., heat to room temperature then cool down to 77 K without laser irradiation (red dashed). (Reprinted with permission from [41]. Copyright 2012 American Chemical Society.)

Thermochromism and optical memory were also observed for Cu(CN)^−^ doped crystals. Between 77 and 200 K, all the peak intensity decreases due to the quenching with increasing temperature. However, above 200 K, the intensity of higher energy peaks abruptly increases and the lower energy peak almost disappears. This is ascribed to the population change during the temperature increasing; the large size nanoclusters break into small size monomers and dimers, which take place between 200 and 250 K. After the reaction is completed, the distribution of nanoclusters no longer changes and the peak intensity is only affected by the temperature. The optical memory experiment was conducted in three steps: (1) the doped crystal is cooled down to liquid nitrogen temperature and during the cooling process, the crystal is irradiated by the 266 nm laser, which is the writing; (2) the emission spectra are recorded at 77 K, the reading; and (3) the crystal is heated up to the room temperature and then cooled down to 77 K again. The luminescence spectra are shown in Figure 9. Excimer and exciplex are more stable and the metal-metal distance is shorter for excited states. The laser irradiation promotes Cu(CN)_2_^−^ ions into an excited state and thus form larger size nanoclusters. With the temperature decrease, they are frozen in their positions, which leads to the distribution change of different nanoclusters.

**Scheme 1:**
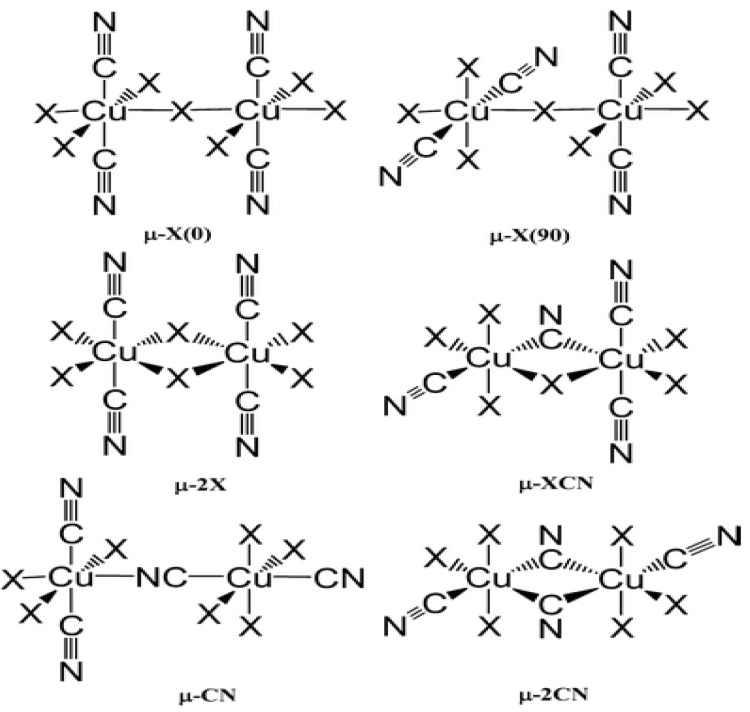
Dimeric configurations of Cu_2_(CN)_4_X_6 or 7_]^8−^ or ^9−^ ions. (Reprinted with permission from [41]. Copyright 2012 American Chemical Society.)

**Figure 10:**
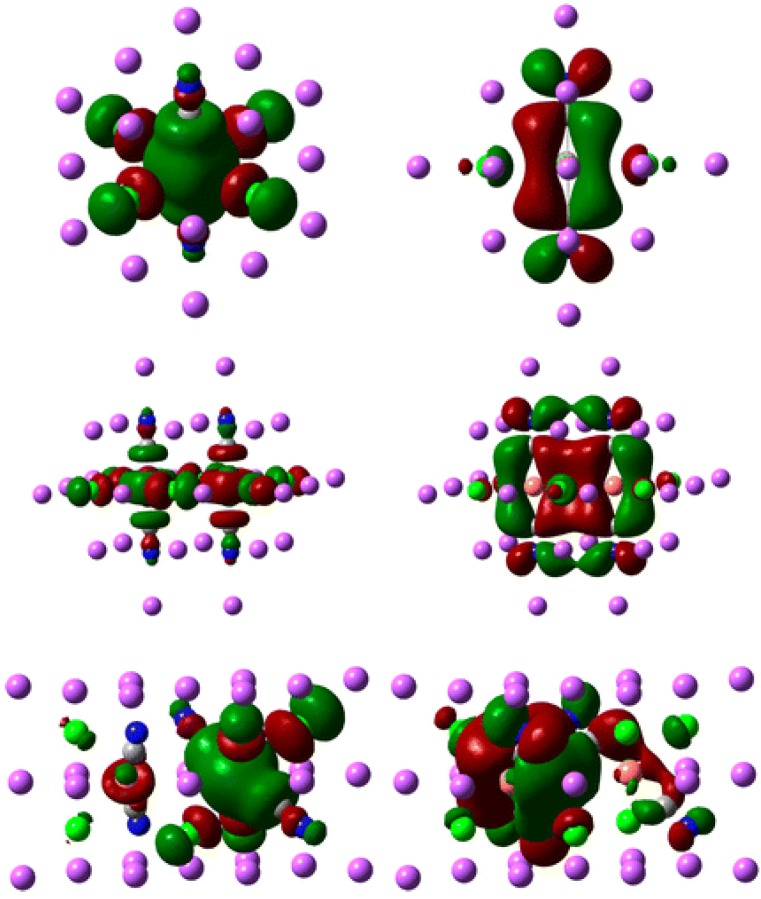
Isodensity of HOMO (left) and LUMO (right) for Cu(CN)_2_^−^ monomers (top), μ-Cl2 dimers (middle) and μ-ClCN dimers (bottom). (Reprinted with permission from [41]. Copyright 2012 American Chemical Society.)

Theoretical density functional calculations were carried out for monomers and possible dimers and the possible configurations are shown in Scheme 1. The calculated electron density results give a major metal-to-ligand charge transfer (MLCT) mixed with ligand-to-ligand charge transfer (LLCT) transition and this is in agreement with the long life time (μs) of all emission peaks, suggesting the phosphorescence process. Figure 10 gives the isodensity map of some of the [Cu(CN)_2_^−^]*_n_* in alkali halide hosts. The optimized structure for monomers and dimers were obtained in the ground state and the first excited triplet state. Comparing the bond distances between the ground state and the first excited triplet state, we find that the Cu–C bond distances contract and the C≡N bond distances expand, which is in accordance with the MLCT transition. Time-dependent DFT calculation results show the absorption and emission energies follow the trend that with the size of nanoclusters increase, the corresponding energies red shift. This is also observed from the experimental results. Atomistic calculations show that the potassium salts have greater bridging state selectivity and more favorable oligomerization energy than that of sodium salts. Bromide salts show a slightly greater bridging state selectivity than chloride salts.

## 5. Conclusions

The dicyano compounds of coinage metals, Ag, Au, and Cu doped in alkali halide hosts show examples of close-shell homometallic interactions, which results in a series of interesting photophysical and photochemical properties. These nanoclusters are typical excimers and exciplexes, e.g., more stable at the excited states. Especially for copper, the Cu(CN)_2_^−^ in alkali halide crystals are unique examples for luminescent anionic Cu(I) compounds. The population of different nanoclusters changes with the concentration, temperature, and laser irradiation. Lifetime results of microsecond magnitude suggest phosphorescence and are consistent with the calculation results, which gives a MLCT transition with slightly LLCT process. The exciplex tuning gives a range as wide as 18,000 cm^−1^ and the strong phosphorescence has possible application for solid state lasers. Thermochromism and optical memory properties can be used to make new photosensors and memory devices.
